# Hourly Heat Exposure and Acute Ischemic Stroke

**DOI:** 10.1001/jamanetworkopen.2024.0627

**Published:** 2024-02-28

**Authors:** Xinlei Zhu, Renjie Chen, Jing Yuan, Yang Liu, Yong Wang, Xunming Ji, Haidong Kan, Jing Zhao

**Affiliations:** 1 School of Public Health, Key Lab of Public Health Safety of the Ministry of Education and National Health Commission Key Lab of Health Technology Assessment, Fudan University, Shanghai, China; 2 Minhang Hospital and School of Pharmacy, Fudan University, Shanghai, China; 3 Department of Neurology, Minhang Hospital, Fudan University, Shanghai, China; 4 Department of Neurosurgery, Xuanwu Hospital, Capital Medical University, Beijing, China

## Abstract

**Importance:**

Previous studies have demonstrated the associations of daily high temperature with hospitalizations and mortality from ischemic stroke, but the hourly association of ambient heat and acute ischemic stroke (AIS) onset has been rarely examined.

**Objectives:**

To evaluate the association between hourly high ambient temperature and the onset of AIS.

**Design, Setting, and Participants:**

This time-stratified case-crossover study was conducted using a nationwide registry that collects data from more than 200 stroke centers in China. Participants were adult patients with AIS who were hospitalized in the warm seasons between January 1, 2019, and December 31, 2021.

**Exposures:**

Hourly temperature and single-hour temperature exposure lag up to 24 hours before the AIS onset (lag 0 hours to lag 24 hours).

**Main Outcomes and Measures:**

The main outcome was onset of AIS. Associations between hourly mean temperatures and AIS onset were analyzed using conditional logistic regression integrated with the distributed lag nonlinear model. Stratification analyses were applied to examine potential association modifiers. Several sensitivity analyses were conducted to examine the robustness of the results.

**Results:**

A total of 82 455 patients with AIS (mean [SD] age, 65.8 [11.9] years; 52 267 males [63.4%]) were included in the final analysis. A monotonically increasing risk of AIS onset was associated with higher temperatures. The excess AIS risk occurred immediately at lag 0 hours and persisted for 10 hours. Compared with the reference temperature (12.1 °C), the cumulative odds ratio (OR) over lag 0 to 10 hours of AIS onset associated with extremely high temperature (33.3 °C) was 1.88 (95% CI, 1.65-2.13) nationwide. The exposure-response curve was steeper in the north than in the south (OR, 1.80 [95% CI, 1.53-2.11] vs 1.57 [95% CI, 1.31-1.87]). The ORs were greater for males and patients with a history of dyslipidemia or atrial fibrillation, but the differences were not significant.

**Conclusions and Relevance:**

Results of this study suggest that hourly heat exposure is associated with increased risk of AIS onset. This finding may benefit the formulation of public health strategies to reduce cerebrovascular risk associated with high ambient temperature under global warming.

## Key Points

**Question:**

Could transient heat exposure at an hourly level trigger the onset of acute ischemic stroke (AIS)?

**Findings:**

In this case-crossover study of 82 455 patients with AIS, there was a monotonically increasing risk of AIS onset associated with higher temperatures. The exposure-response curve was steeper in the northern region than in the southern region.

**Meaning:**

Findings of this study may benefit the formulation of public health strategies to reduce the cerebrovascular risk associated with high ambient temperature under global warming.

## Teaser

This case-crossover study evaluates the risk of acute ischemic stroke amid hourly exposure to hot temperature during warmer seasons among adults in China.

## Introduction

Stroke is a substantial factor in global mortality and disability, with incidence, prevalence, disability-adjusted life years, and excess deaths increasing over the past decades.^1^ According to the 2019 Global Burden of Disease Study, the number of ischemic stroke cases in China for 2019 reached 2.87 million, accounting for approximately 72.8% of the total incident cases of stroke.^2^ Given that acute ischemic stroke (AIS) is a severe medical condition that leads to serious disability and death,^3^ identifying its modifiable risk factors is crucial for clinical practice and public health. Established risk factors for AIS include hypertension, hypercholesterolemia, cigarette smoking, poor nutrition, physical inactivity, and air pollution. As climate change continues to be a pressing public health challenge,^4^ there is an urgent need to better understand the association between warm weather conditions and stroke incidence.

Several observational studies have reported an approximately linear association between heat exposure and stroke risk.^5,6,7,8,9^ However, most of these studies focused on daily hospitalization or mortality, which could not accurately reflect the time of disease onset and could have resulted in chronological disorder between temperature exposure and AIS onset. Additionally, most prior studies applied a time series approach, which used the daily counts of AIS hospital admissions^8,10,11^ and hence could lead to apparent ecological bias and weakened causality.^12^ Furthermore, some case-crossover studies encountered constraints due to their relatively limited sample sizes and confined geographical scopes (eg, single center).^5,13,14^ To date, only 1 multicenter study has investigated the associations between ambient heat and AIS at an hourly level.^15^ However, that study applied the delay time prior to hospitalization instead of the specific AIS onset time for exposure matching, resulting in inevitable exposure misclassification. Furthermore, although prior evidence suggested that heat-related AIS is more likely to occur at the subdaily level,^14,16,17^ few studies have directly evaluated the sensitive time windows at the hourly level, which is helpful for designing heat-related warning systems.^7,13^ Thus, a nationwide case-crossover investigation based on individual AIS onset time is imperative to quantify the association between AIS onset and hourly ambient heat exposure.

As one of the world’s largest middle-income countries, China has the highest stroke burden^18^ and is among the most vulnerable to global climate change.^19^ Accordingly, in the present time-stratified case-crossover analysis, we aimed to evaluate the association between hourly high ambient temperature and the onset of AIS using a nationwide registry of AIS. Additionally, we assessed possible association modifiers, such as geographic region, smoking, alcohol drinking, sex, age, and history of other disease.

## Methods

### Study Population and Outcome Data

Data pertaining to the onset of AIS were extracted from the Bigdata Observatory Platform for Stroke of China (BOSC), which collects data from more than 200 stroke centers in 31 provinces across mainland China. Structured validation procedures are performed periodically to check the completeness and plausibility of these data. A comprehensive description of the BOSC has been published in prior studies.^20,21^ The BOSC includes demographic characteristics, diagnoses, severity assessments, treatment modalities, and complications. The Minhang Hospital at Fudan University Institutional Review Board approved the study and waived the informed consent requirement because only deidentified data were used in the analysis. We followed the Strengthening the Reporting of Observational Studies in Epidemiology (STROBE) reporting guideline.

The AIS diagnosis was made using the *International Statistical Classification of Diseases and Related Health Problems, Tenth Revision,* diagnosis code I63. For this analysis, we included patients with AIS who met the following criteria: (1) age 18 years or older; (2) hospital admission within 7 days after the hour of self-reported symptom onset to reduce the likelihood of recall bias; and (3) hospitalization in the warm season (from April 1 to September 30) between January 1, 2019, and December 31, 2021. Patients who did not report the detailed time of AIS symptom onset were excluded.

### Study Design

This observational study has a time-stratified case-crossover design, which is a case-only approach that uses each case as its own control. Thus, this design could effectively control for confounders that are time invariant or exhibit stability within a short period, such as comorbid, demographic, and behavioral risk factors. The hour of self-reported AIS onset was defined as the case hour. For every case hour, the matching process ensued, encompassing 3 or 4 control hours characterized by identical attributes of the same year, month, day of the week, and hour of the day, thereby controlling for long-term variations, seasonality, and circadian rhythms. For example, if the first AIS symptom occurred at 8 am on Tuesday, May 19, 2020, we would define 8 am on Tuesday, May 19, 2020, as the case hour and 8 am on all other Tuesdays in May 2020 (May 5, 12, and 26) as the control hours.

### Exposure Data

We obtained the data on hourly temperature and relative humidity from the nearest weather stations affiliated with the China Meteorological Data Sharing Service System. We excluded hourly temperatures below the first percentile or above the 99th percentile to minimize the implications of extreme values. We matched hourly meteorological data from the nearest weather stations according to patients’ residential addresses. To reduce exposure measurement errors, we considered for inclusion only the addresses situated within 100 km of the weather station. We defined exposure at lag 0 hours as the temperature during the preceding natural hour if the event occurred in the first half of the index hour or the temperature at the concurrent natural hour if it occurred in the second half of the index hour. Accordingly, we evaluated single-hour lags up to 24 hours before the AIS onset (lag 0 hours to lag 24 hours).

To account for the implications of air pollution, we further collected through the nearest station the hourly concentrations of fine particulate matter, nitrogen dioxide, sulfur dioxide, ozone, and carbon monoxide from the National Urban Air Quality Real-time Publishing Platform. We restricted the assignment of air pollution data to patients’ home addresses within a 50-km radius of the nearest monitoring stations.

### Statistical Analysis

The association between hourly exposures to AIS onset and ambient temperature was analyzed through the application of a conditional logistic regression model in conjunction with the distributed lag nonlinear model. This modeling approach enabled the exploration of potential nonlinear and cumulative exposure-response associations across various lag periods.^22^ We created a cross-basis function by merging 2 sets of basic functions, one describing exposure structure and the other representing lag structure with a natural cubic spline, with 2 and 3 degrees of freedom (*df*), respectively. Specifically, a natural cubic spline with 1 internal knot was fitted to account for potential nonlinear associations between temperature and AIS onset. For the lags, a natural cubic spline with 2 internal knots was selected at equally spaced log values of lags to allow for more flexibility at shorter delays.^22^ Given that previous studies have shown that the association of high temperature with AIS mainly occurred within 1 day,^7,23^ we selected a priori a maximum lag of 24 hours in the distributed lag nonlinear model. Subsequently, the cross-basis function was incorporated into the model, adjusting for a binary variable indicating public holidays and a natural cubic spline (*df = *3) for the 0 to 24 hours’ mean of relative humidity. The temperature corresponding to the lowest AIS risk was used as the reference. We illustrated the lag pattern for the odds ratios (ORs) by comparing the extremely high temperature (99th percentile of the temperature distribution)^24,25,26,27^ with the reference. By visualizing the lag pattern, we determined the lag that generated the largest risk, which was then used to estimate the cumulative associations and to plot the exposure-response curves. We further computed ORs and 95% CIs of AIS by comparing the extremely high temperatures (99th percentile of the temperature distribution) with the reference.

To investigate the potential regional disparities, we developed separate models for cases originating from the southern and northern regions demarcated by the established Qinling-Huaihe Line. Furthermore, stratified analyses were undertaken, encompassing parameters, such as sex (male or female), smoking (yes or no), alcohol drinking (yes or no), age (<65 years or ≥65 years), and history of other diseases (hypertension, dyslipidemia, and atrial fibrillation), to explore possible individual-level association modifiers. Stratum-specific estimates were compared using *z* tests based on the corresponding point estimates and their SEs.^28^ A total of 7 sensitivity analyses were conducted to evaluate the robustness of the findings (eMethods in Supplement 1).

All statistical analyses were conducted using R, version 3.4.2 (R Project for Statistical Computing). The tests were 2-sided, and statistical significance was determined at α < .05.

## Results

### Descriptive Results

After exclusions, 82 455 patients with AIS from 329 cities were included for the final analysis (eFigures 1 and 2 in Supplement 1). These patients had a mean (SD) age of 65.8 (11.9) years and included 30 188 females (36.6%) and 52 267 males (63.4%), of whom 56.5% resided in the northern region and 43.5% in the southern region (Table 1). Furthermore, 32.1% had a smoking status, 24.2% had an alcohol drinking status, and 66.1% had a history of disease (hypertension, dyslipidemia, or atrial fibrillation). The proportion of 3-hour hospital arrivals after symptom onset was 11.8%. The mean (SD) temperature and humidity from lag 0 to 24 hours during the study period were 24.2 (4.1) °C and 75.1% (14.6%) (Table 2).

**Table 1.** Summary of the Associations Between Acute Ischemic Stroke and Extremely High Temperature^a^

Characteristic	Case, No. (%)	Temperature, °C		OR (95% CI)	*P* value for z score
		Extremely high	Reference		
National level	82 455 (100.0)	33.3	12.1	1.88 (1.65-2.13)	NA
Regional level					
North	46 550 (56.5)	32.0	10.2	1.80 (1.53-2.11)	.27
South	35 905 (43.5)	34.2	17.1	1.57 (1.31-1.87)	
Sex					
Male	52 267 (63.4)	33.3	12.0	1.96 (1.67-2.31)	.34
Female	30 188 (36.6)	33.3	12.2	1.72 (1.39-2.13)	
Age, y^b^					
≥65	46 446 (56.3)	33.4	12.3	1.83 (1.54-2.17)	.76
<65	35 985 (43.6)	33.2	11.9	1.91 (1.57-2.32)	
Smoking^c^					
Yes	26 497 (32.1)	33.2	11.8	1.96 (1.56-2.45)	.55
No	55 950 (67.9)	33.4	12.3	1.80 (1.54-2.10)	
Alcohol drinking^d^					
Yes	19 981 (24.2)	33.2	11.8	1.83 (1.25-2.13)	.67
No	62 463 (75.8)	33.4	12.2	1.96 (1.69-2.27)	
History of other disease^e^					
No history of other disease	27 952 (33.9)	33.2	12.1	1.94 (1.58-2.38)	NA
History of hypertension	49 259 (59.7)	33.4	12.2	1.80 (1.53-2.12)	.59^f^
History of dyslipidemia	2433 (3.0)	33.2	12.1	4.68 (2.15-10.17)	.68^f^
History of atrial fibrillation	2794 (3.4)	33.5	12.6	2.17 (0.86-4.34)	.80^f^

Abbreviations: NA, not applicable; OR, odds ratio.

^a^ The associations are presented as cumulative ORs comparing the extremely high temperature (99th percentile of temperature distribution) to the reference temperature (first percentile of temperature distribution) over lag 0 to 10 hours.

^b^ Missing data for 24 cases.

^c^ Missing data for 8 cases.

^d^ Missing data for 11 cases.

^e^ Missing data for 17 cases.

^f^ Each *P* value was calculated for testing the difference between the group No history of other disease and 1 of the other 3 groups.

**Table 2.** Descriptive Statistics of Environmental Data^a^

Variables	Mean (SD)	Percentile of temperature distribution				
		1st	25th	50th	75th	99th
Weather conditions						
Temperature, °C	24.2 (4.1)	18.3	21.6	24.7	27.2	30.9
Relative humidity, %	75.1 (14.6)	55.8	67.3	77.0	85.5	98.9
Pollutants						
PM_2.5_, μg/m^3^	22.6 (13.7)	8.3	12.7	19.8	29.6	65.8
NO_2_, μg/m^3^	21.3 (11.0)	9.4	13.3	19.3	27.1	55.7
SO_2_, μg/m^3^	8.0 (4.9)	3.3	5.0	6.9	9.8	25.1
O_3_, μg/m^3^	72.9 (30.3)	36.4	50.3	69.4	92.0	154.0
CO, mg/m^3^	0.7 (0.3)	0.4	0.5	0.6	0.8	1.4

Abbreviations: CO, carbon monoxide; NO_2_, nitrogen dioxide; O_3_, ozone; PM_2.5_, fine particulate matter; SO_2_, sulfur dioxide.

^a^ The descriptive statistics of environmental data were calculated on the basis of moving backward 24 hours from the onset hour of acute ischemic stroke.

### Associations Between High Temperature and AIS Onset

**Figure 1.**
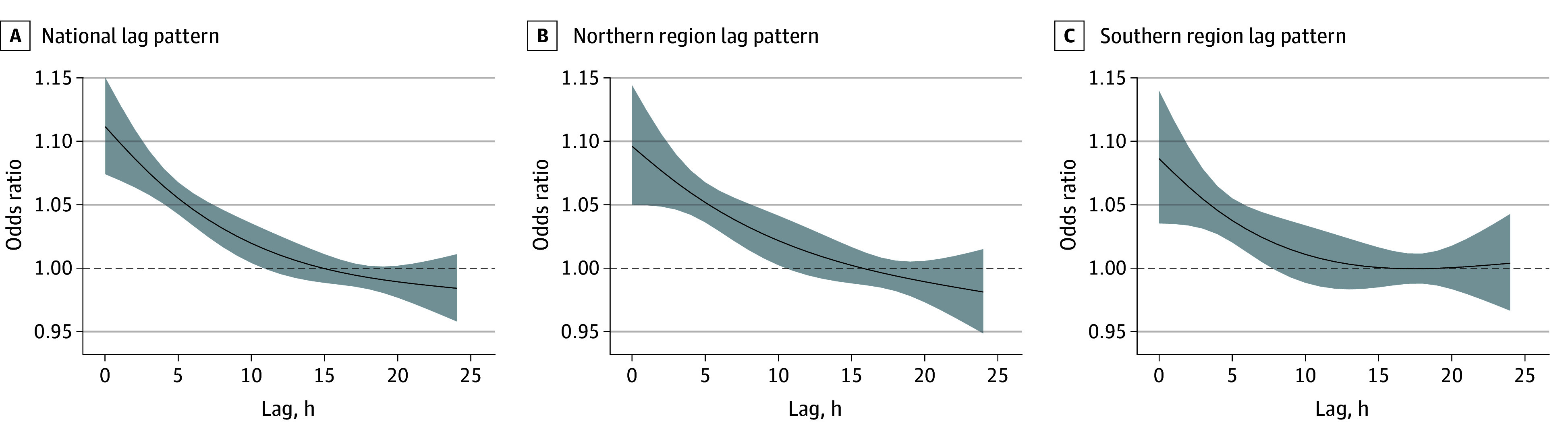
National and Regional Lag Patterns for the Association Between Acute Ischemic Stroke (AIS) Onset and Extremely High Temperature The curved lines indicate the odds ratios of AIS onset; shaded areas, 95% CIs.

Figure 1 depicts the lag patterns for ORs of AIS onset at extreme high temperatures compared with the reference temperatures, both nationally and regionally. At the national level (Figure 1A), the association between high temperatures and AIS onset was evident at the concurrent hour of exposure (OR, 1.11; 95% CI, 1.07-1.15), attenuated over time, and became insignificant approximately at lag 10 hours (OR, 1.02; 95% CI, 1.00-1.04). At the regional level (Figure 1B and C), the southern and northern regions exhibited lag patterns similar to those of the national estimate.

**Figure 2.**
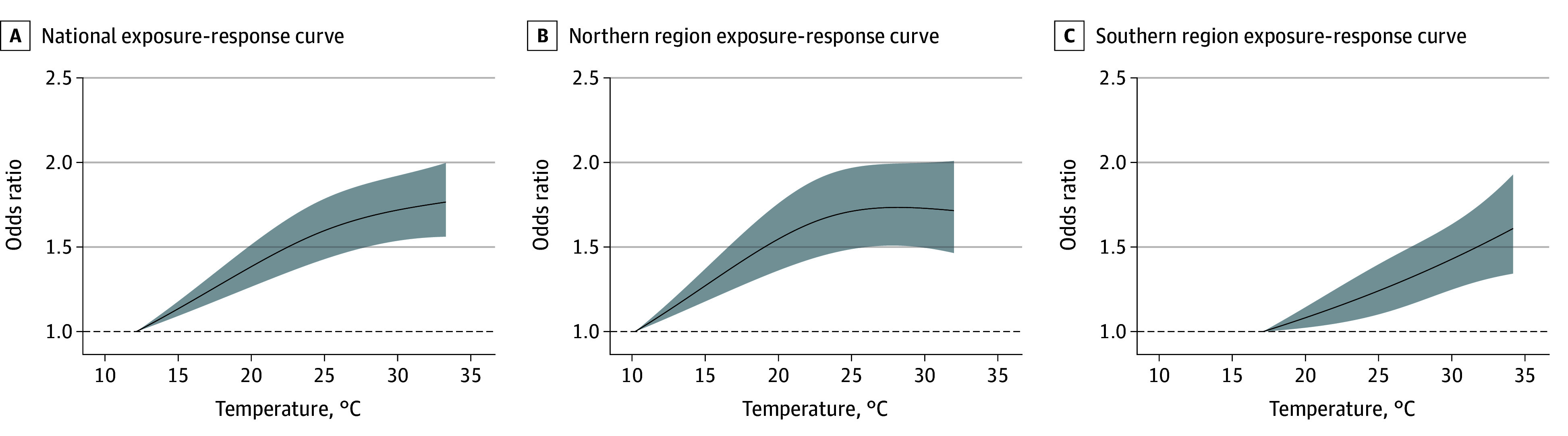
National and Regional Exposure-Response Curves for the Association Between Ambient Temperature and Acute Ischemic Stroke Onset Accumulated Over 0 to 10 Hours The curved lines indicate the odds ratios of AIS onset; shaded areas, 95% CIs.

Subsequently, we plotted the cumulative exposure-response curves for the association between high temperature and AIS onset over lags 0 to 10 hours (Figure 2). The risk of AIS onset was monotonically increasing with higher temperatures and tended to level off at extremely high temperatures. The exposure-response curve was steeper in the northern region than in the southern region (OR, 1.80 [95% CI, 1.53-2.11] vs 1.57 [95% CI, 1.31-1.87]) (Table 1), and the apparent leveling off occurred at higher temperatures in the north.

Given the almost linear association between high temperature and AIS onset, we defined the extremely low temperature (first percentile of the temperature distribution) as the reference temperature and computed ORs and 95% CIs of AIS by comparing the extremely high temperatures with the reference. Table 1 summarizes the cumulative associations over lags 0 to 10 hours between extremely high temperature and AIS onset. At the national level, comparing the extremely high temperature (33.3 °C) with the reference temperature (12.1 °C), the cumulative OR of AIS episodes was 1.88 (95% CI, 1.65-2.13). The OR was higher in the north than in the south (1.80 [95% CI, 1.53-2.11] vs 1.57 [95% CI, 1.31-1.87]; *P* = .27).

We found a higher risk of AIS associated with high temperature among males than among females in stratified analyses, whereas the association estimates in patients 65 years or older vs younger than 65 years were quite similar, as were estimates in those with vs without smoking and drinking (Table 1). Moreover, those with a history of dyslipidemia had a higher OR (4.68; 95% CI, 2.15-10.17) than those with a history of hypertension (1.80; 95% CI, 1.53-2.12) and atrial fibrillation (2.17; 95% CI, 0.86-4.34). However, the differences between these subgroups were not statistically significant (Table 1). The sensitivity analyses revealed the robustness of these results (eFigures 3 and 4 in Supplement 1; Table 3). For example, the main estimates remained stable after altering the *df* of cross-basis functions (OR, 1.83; 95% CI, 1.55-2.17), restricting the distance between the home address and the nearest weather station within 50 km (OR, 1.90; 95% CI, 1.67-2.17) and adjusting for air pollutants (OR, 1.53; 95% CI, 1.35-1.73).

**Table 3.** Associations Between Acute Ischemic Stroke and Extremely High Temperature in Multiple Sensitivity Analyses^a^

Variable	OR (95% CI)		
	National	Northern region	Southern region
Main analysis (n = 82 455)^b^	1.88 (1.65-2.13)	1.80 (1.53-2.11)	1.57 (1.31-1.87)
Sensitivity analysis			
Distance: ≤50 km (n = 79 870)^c^	1.90 (1.67-2.17)	1.84 (1.56-2.18)	1.57 (1.31-1.88)
*df* Of natural cubic spline: 3 (n = 82 455)^d^	1.83 (1.55-2.17)	1.77 (1.43-2.19)	1.63 (1.27-2.08)
Maximum lag period: 36 h (n = 82 455)	1.83 (1.59-2.11)	1.79 (1.49-2.15)	1.48 (1.22-1.79)
Different quantiles of high temperatures (n = 82 455)			
95th Percentile of temperature distribution: 31.9 °C	1.83 (1.62-2.07)	1.73 (1.48-2.02)	1.53 (1.30-1.81)
90th Percentile of temperature distribution: 30.5 °C	1.79 (1.59-2.02)	1.67 (1.44-1.94)	1.50 (1.28-1.76)
80th Percentile of temperature distribution: 28.6 °C	1.73 (1.54-1.95)	1.58 (1.37-1.83)	1.45 (1.24-1.69)
70th Percentile of temperature distribution: 27.1 °C	1.68 (1.50-1.89)	1.52 (1.32-1.76)	1.41 (1.21-1.63)
60th Percentile of temperature distribution: 25.8 °C	1.63 (1.46-1.83)	1.47 (1.28-1.69)	1.37 (1.18-1.58)
50th Percentile of temperature distribution: 24.5 °C	1.58 (1.42-1.76)	1.42 (1.24-1.62)	1.33 (1.16-1.53)
Analysis among cases with pollutant data (n = 82 455)			
+ PM_2.5_	1.68 (1.48-1.90)	1.61 (1.38-1.89)	1.38 (1.17-1.62)
+ SO_2_	1.66 (1.47-1.88)	1.61 (1.37-1.88)	1.38 (1.17-1.62)
+ NO_2_	1.65 (1.46-1.86)	1.58 (1.35-1.85)	1.38 (1.17-1.62)
+ CO	1.67 (1.48-1.89)	1.61 (1.38-1.89)	1.38 (1.17-1.62)
+ O_3_	1.66 (1.47-1.88)	1.63 (1.40-1.91)	1.36 (1.16-1.60)
+ All 5 pollutants	1.53 (1.35-1.73)	1.52 (1.30-1.79)	1.26 (1.07-1.48)

Abbreviations: *df*, degrees of freedom; CO, carbon monoxide; NO_2_, nitrogen dioxide; O_3_, ozone; OR, odds ratio; PM_2.5_, fine particulate matter; SO_2_, sulfur dioxide.

^a^ The associations are presented as cumulative ORs of acute ischemic stroke onset comparing the extremely high temperatures to the reference temperatures over lag 0 to 10 hours, which are provided in Table 2.

^b^ Settings of main analysis: (1) 99th percentile of the temperature distribution (33.2 °C); distance of 100 km or less; (3) *df* of natural cubic spline of 2; and (4) maximum lag period of 24 hours.

^c^ Distance refers to the distance between patient addresses and the nearby weather station.

^d^ Natural cubic spline was applied in cross-basis function to describe exposure structure.

## Discussion

Findings of this case-crossover study suggest that a transient exposure to high temperature could be a factor in the onset of AIS episodes, with the association occurring at the concurrent hour and lasting for up to 10 hours. The association was greater in magnitude in the north than in the south. Furthermore, we observed higher ORs among male patients and those with a history of dyslipidemia and atrial fibrillation, although the findings were not statistically significant.

The use of individual-level, hourly information on AIS onset is a strength of this study. Prior investigations have typically relied on time-series analyses of aggregate cases of stroke deaths or hospitalizations and exposure data at the daily timescale,^29,30^ which would result in concerns about ecological fallacy, exposure misclassification, and the unclear time window most relevant to the onset of AIS on the subdaily basis. While some case-crossover studies had been conducted on a daily scale, they had estimated association values that were smaller in magnitude compared with the present study.^7,14^ It is likely that heat exposure has more immediate associations that could occur on the subdaily basis, and estimates on the daily basis may be attenuated when the mean exposures over the day were calculated. We consistently observed higher ORs for acute heat exposure in lag 0 hours, indicating that the risk of AIS associated with extremely high temperature may not be long lasting.

To our knowledge, 2 other case-crossover studies have investigated these associations at the hourly timescale. One study that was conducted by Vodonos et al^11^ in a medical center and involved 1147 patients reported an association between high temperature and incidence of ischemic stroke. The lag time of the association in that study extended up to 96 hours, which is longer than in the present study. The smaller sample size and single-center design of the study by Vodonos et al^11^ might account for the observed differences from the present findings. Similar exposure time windows were identified in another multicenter study conducted in the US, which included 578 181 patients with ischemic stroke.^15^ The study by Rowland et al^15^ found that higher temperature within a 7-hour period after exposure was associated with an increase in stroke onset risk, with a 10 °C temperature increase over 7 hours corresponding to a 5.1% (95% CI, 3.8%-6.4%) increase in stroke onset risk. In comparison, the associations observed in the present study were greater in magnitude. The exposure assessment in Rowland et al^15^ assumed a 3-hour prehospital delay for all patients. However, according to the BOSC dataset, the proportion of 3-hour hospital arrivals after symptom onset was only 11.8%. Therefore, only considering a 3-hour prehospital delay for all patients could lead to a considerable underestimation of the associations. In contrast, we used self-reported onset times for all patients, thereby minimizing the bias associated with the use of assumed prehospital delay times as the AIS onset time.

The association of high temperature with AIS onset was greater in magnitude in the north than in the south. This regional discrepancy may be due to the better heat adaptability for populations in the south where the climate was warmer, which is consistent with previous findings.^31,32^ Populations in the northern region, who may be less accustomed to high temperatures, may display increased sensitivity, as evidenced by a steeper exposure-response curve. Furthermore, we identified a higher vulnerability in males, potentially associated with more outdoor activities and higher baseline prevalence of stroke.^9,11,16^ Additionally, we observed elevated risks among patients with preexisting hyperlipidemia and atrial fibrillation, which are plausible findings, as these diseases are established risk factors for AIS.^33,34,35^ It is still imperative for public health professionals to disseminate pertinent information regarding disease prevention and health promotion to at-risk populations, thereby increasing their heat adaptation ability.^36^ Patients, particularly males with dyslipidemia or atrial fibrillation, should exercise with caution, reduce outdoor activities, and use air conditioning to protect themselves from high temperatures.

The biological mechanisms underlying the association between high ambient temperature and AIS onset remain to be elucidated. It is hypothesized that heat exposure may elevate the risk of ischemic stroke or transient ischemic attack by inducing thromboembolism from hemoconcentration and hyperviscosity.^37^ Heat exposure could increase skin blood flow and sweating, leading to dehydration and consequent hemoconcentration and hyperviscosity. These factors may be associated with the formation of thromboembolism and thereby with the increased risk of ischemic stroke.^16^ The increasing gut epithelial membrane permeability due to the blood flow redistribution allows more bacteria to get into the systemic circulation and release different endotoxins and lipopolysaccharides, which leads to the syndrome of systemic inflammatory response.^38^ Together with the endothelium dysfunction related to thermal stress,^39^ vascular malfunction might play a role in plaque instability, increasing the risk of AIS onset.

### Strengths and Limitations

This study has several strengths. First, the use of the BOSC data, which covers hundreds of major hospitals across China, ensured high data quality and spatial representativeness. The ample individual information for patients with AIS also enabled us to explore the potentially at-risk populations. Second, this time-stratified case-crossover study was conducted at the individual level, which could autonomically control for time-invariant confounders and thus may facilitate the causal inferences unlike with previous ecological time-series studies. Third, by virtue of the hourly information on AIS onset, we could characterize the subdaily time course from heat exposure to onset of AIS symptoms, which can be helpful in tailoring a heat warning system.

Nevertheless, this study has several limitations. First, as in many previous studies, in this study temperature exposures were estimated using nearby fixed-site monitors, leading to inevitable exposure misclassification. However, temperature is largely homogeneous within a relatively broad area, and any resulting errors may be random and may have led to an underestimation of the true associations.^40^ Additionally, we conducted a sensitivity analysis by restricting the distance to the weather stations and observed similar results. Second, in such a nationwide study, there could be inevitable recall bias in self-reported onset time of AIS. We minimized this error by excluding patients with self-reported time of onset beyond 7 days before hospital admission. Third, the study exclusively encompassed patients with AIS admitted to hospitals, excluding those who succumbed to AIS prior to hospital admission. Consequently, if high temperature exhibits an association with AIS survival, there is a possibility that the true association between temperature and AIS onset could be underestimated.

## Conclusions

This national case-crossover study in China found an association between hourly exposure to high temperatures and the onset of AIS. It presents robust evidence of high temperature as an important factor in increased risk of AIS onset. The results add valuable insights to the adverse cardiovascular outcome of climate warming and may benefit the formulation of public health strategies to reduce cerebrovascular risk associated with high ambient temperature under global warming. Additionally, the findings underscore the ongoing need for public health agencies to advocate for interventions that mitigate heat exposure and bolster cooling measures, particularly among populations at high risk for AIS.

## Notes

Supplement 1. **eMethods.** Sensitivity Analysis **eFigure 1.** Flowchart for the Inclusion and Exclusion of AIS Patients **eFigure 2.** Geographic Distribution of the AIS Patients Included in This Study in China **eFigure 3.** National and Region-Specific Exposure-Response Curves for the Associations of AIS Onset With Extremely High Temperature From the Analysis With 3 Degrees of Freedom for the Natural Cubic Spline Applied in the Main Models **eFigure 4.** National and Region-Specific Lag Structures for the Associations of AIS Onset With Extremely High Temperature From the Analysis With the Maximum Lag of 36 Hours

Supplement 2.
Data Sharing Statement

## References

[1] Feigin VL, Forouzanfar MH, Krishnamurthi R, ; Global Burden of Diseases, Injuries, and Risk Factors Study 2010 (GBD 2010) and the GBD Stroke Experts Group. Global and regional burden of stroke during 1990-2010: findings from the Global Burden of Disease Study 2010. Lancet. 2014;383(9913):245-254. doi:10.1016/S0140-6736(13)61953-4 24449944 PMC4181600

[2] Ma Q, Li R, Wang L, . Temporal trend and attributable risk factors of stroke burden in China, 1990-2019: an analysis for the Global Burden of Disease Study 2019. Lancet Public Health. 2021;6(12):e897-e906. doi:10.1016/S2468-2667(21)00228-0 34838196 PMC9047702

[3] Mendelson SJ, Prabhakaran S. Diagnosis and management of transient ischemic attack and acute ischemic stroke: a review. JAMA. 2021;325(11):1088-1098. doi:10.1001/jama.2020.26867 33724327

[4] Costello A, Abbas M, Allen A, . Managing the health effects of climate change: Lancet and University College London Institute for Global Health Commission. Lancet. 2009;373(9676):1693-1733. doi:10.1016/S0140-6736(09)60935-1 19447250

[5] Chen JH, Jiang H, Wu L, . Association of ischemic and hemorrhagic strokes hospital admission with extreme temperature in Nanchang, China-a case-crossover study. J Clin Neurosci. 2017;43:89-93. doi:10.1016/j.jocn.2017.04.044 28629681

[6] Wang X, Cao Y, Hong D, . Ambient temperature and stroke occurrence: a systematic review and meta-analysis. Int J Environ Res Public Health. 2016;13(7):698. doi:10.3390/ijerph13070698 27420077 PMC4962239

[7] Vered S, Paz S, Negev M, Tanne D, Zucker I, Weinstein G. High ambient temperature in summer and risk of stroke or transient ischemic attack: a national study in Israel. Environ Res. 2020;187:109678. doi:10.1016/j.envres.2020.109678 32474306

[8] Wang Q, Gao C, Wang H, Lang L, Yue T, Lin H. Ischemic stroke hospital admission associated with ambient temperature in Jinan, China. PLoS One. 2013;8(11):e80381. doi:10.1371/journal.pone.0080381 24260379 PMC3833907

[9] Lian H, Ruan Y, Liang R, Liu X, Fan Z. Short-term effect of ambient temperature and the risk of stroke: a systematic review and meta-analysis. Int J Environ Res Public Health. 2015;12(8):9068-9088. doi:10.3390/ijerph120809068 26264018 PMC4555265

[10] Tian Y, Liu H, Zhao Z, . Association between ambient air pollution and daily hospital admissions for ischemic stroke: a nationwide time-series analysis. PLoS Med. 2018;15(10):e1002668. doi:10.1371/journal.pmed.1002668 30286080 PMC6171821

[11] Vodonos A, Novack V, Horev A, Abu Salameh I, Lotan Y, Ifergane G. Do gender and season modify the triggering effect of ambient temperature on ischemic stroke? Womens Health Issues. 2017;27(2):245-251. doi:10.1016/j.whi.2016.11.002 28007390

[12] Chen R, Jiang Y, Hu J, . Hourly air pollutants and acute coronary syndrome onset in 1.29 million patients. Circulation. 2022;145(24):1749-1760. doi:10.1161/CIRCULATIONAHA.121.057179 35450432

[13] Gomes J, Damasceno A, Carrilho C, . Triggering of stroke by ambient temperature variation: a case-crossover study in Maputo, Mozambique. Clin Neurol Neurosurg. 2015;129:72-77. doi:10.1016/j.clineuro.2014.12.002 25559679 PMC4339044

[14] Rakers F, Schiffner R, Rupprecht S, . Rapid weather changes are associated with increased ischemic stroke risk: a case-crossover study. Eur J Epidemiol. 2016;31(2):137-146. doi:10.1007/s10654-015-0060-3 26148559

[15] Rowland ST, Chillrud LG, Boehme AK, . Can weather help explain ‘why now?’: the potential role of hourly temperature as a stroke trigger. Environ Res. 2022;207:112229. doi:10.1016/j.envres.2021.112229 34699760 PMC8810591

[16] Lavados PM, Olavarría VV, Hoffmeister L. Ambient temperature and stroke risk: evidence supporting a short-term effect at a population level from acute environmental exposures. Stroke. 2018;49(1):255-261. doi:10.1161/STROKEAHA.117.017838 29229725

[17] Zhang YQ, Yu CH, Bao JZ. Impact of daily mean temperature, cold spells, and heat waves on stroke mortality a multivariable Meta-analysis from 12 counties of Hubei province, China [in Chinese]. Zhonghua Liu Xing Bing Xue Za Zhi. 2017;38(4):508-513. 28468072 10.3760/cma.j.issn.0254-6450.2017.04.019

[18] Wang W, Jiang B, Sun H, ; NESS-China Investigators. Prevalence, incidence, and mortality of stroke in China: results from a nationwide population-based survey of 480 687 adults. Circulation. 2017;135(8):759-771. doi:10.1161/CIRCULATIONAHA.116.025250 28052979

[19] Chan EYY, Ho JY, Hung HHY, Liu S, Lam HCY. Health impact of climate change in cities of middle-income countries: the case of China. Br Med Bull. 2019;130(1):5-24. doi:10.1093/bmb/ldz011 31070715 PMC6587073

[20] Tu WJ, Chao BH, Yan F, Cao L, Wang LD. Stroke unit care for ischemic stroke in China: results of a nation-based study. Intensive Care Med. 2020;46(7):1489-1491. doi:10.1007/s00134-020-06046-x 32338307

[21] Shen Y, Chao BH, Cao L, Tu WJ, Wang LD. Stroke center care and outcome: results from the CSPPC stroke program. Transl Stroke Res. 2020;11(3):377-386. doi:10.1007/s12975-019-00727-6 31494833

[22] Guo Y, Barnett AG, Pan X, Yu W, Tong S. The impact of temperature on mortality in Tianjin, China: a case-crossover design with a distributed lag nonlinear model. Environ Health Perspect. 2011;119(12):1719-1725. doi:10.1289/ehp.1103598 21827978 PMC3261984

[23] Lei L, Bao J, Guo Y, Wang Q, Peng J, Huang C. Effects of diurnal temperature range on first-ever strokes in different seasons: a time-series study in Shenzhen, China. BMJ Open. 2020;10(11):e033571. doi:10.1136/bmjopen-2019-033571 33444167 PMC7682471

[24] Andhikaputra G, Sapkota A, Lin YK, . The impact of temperature and precipitation on all-infectious-, bacterial-, and viral-diarrheal disease in Taiwan. Sci Total Environ. 2023;862:160850. doi:10.1016/j.scitotenv.2022.160850 36526204

[25] Yang J, Yin P, Zhou M, . The effect of ambient temperature on diabetes mortality in China: A multi-city time series study. Sci Total Environ. 2016;543(Pt A):75-82. doi:10.1016/j.scitotenv.2015.11.014 26580729

[26] Zhang H, Wang Q, Benmarhnia T, . Assessing the effects of non-optimal temperature on risk of gestational diabetes mellitus in a cohort of pregnant women in Guangzhou, China. Environ Int. 2021;152:106457. doi:10.1016/j.envint.2021.106457 33706037

[27] Alahmad B, Khraishah H, Royé D, . Associations between extreme temperatures and cardiovascular cause-specific mortality: results from 27 countries. Circulation. 2023;147(1):35-46. doi:10.1161/CIRCULATIONAHA.122.061832 36503273 PMC9794133

[28] Liu Y, Pan J, Fan C, . Short-term exposure to ambient air pollution and mortality from myocardial infarction. J Am Coll Cardiol. 2021;77(3):271-281. doi:10.1016/j.jacc.2020.11.033 33478650

[29] Lokken RP, Wellenius GA, Coull BA, . Air pollution and risk of stroke: underestimation of effect due to misclassification of time of event onset. Epidemiology. 2009;20(1):137-142. doi:10.1097/EDE.0b013e31818ef34a 19244659 PMC2888684

[30] Zhang Y, Li C, Feng R, . The short-term effect of ambient temperature on mortality in Wuhan, China: a time-series study using a distributed lag non-linear model. Int J Environ Res Public Health. 2016;13(7):722. doi:10.3390/ijerph13070722 27438847 PMC4962263

[31] Moghadamnia MT, Ardalan A, Mesdaghinia A, Keshtkar A, Naddafi K, Yekaninejad MS. Ambient temperature and cardiovascular mortality: a systematic review and meta-analysis. PeerJ. 2017;5:e3574. doi:10.7717/peerj.3574 28791197 PMC5546177

[32] He F, Wei J, Dong Y, . Associations of ambient temperature with mortality for ischemic and hemorrhagic stroke and the modification effects of greenness in Shandong Province, China. Sci Total Environ. 2022;851(Pt 1):158046. doi:10.1016/j.scitotenv.2022.158046 35987239

[33] Migdady I, Russman A, Buletko AB. Atrial fibrillation and ischemic stroke: a clinical review. Semin Neurol. 2021;41(4):348-364. doi:10.1055/s-0041-1726332 33851396

[34] Aigner A, Grittner U, Rolfs A, Norrving B, Siegerink B, Busch MA. Contribution of established stroke risk factors to the burden of stroke in young adults. Stroke. 2017;48(7):1744-1751. doi:10.1161/STROKEAHA.117.016599 28619986

[35] Healey JS, Amit G, Field TS. Atrial fibrillation and stroke: how much atrial fibrillation is enough to cause a stroke? Curr Opin Neurol. 2020;33(1):17-23. doi:10.1097/WCO.0000000000000780 31809335

[36] Kreslake JM, Sarfaty M, Roser-Renouf C, Leiserowitz AA, Maibach EW. The critical roles of health professionals in climate change prevention and preparedness. Am J Public Health. 2018;108(S2):S68-S69. doi:10.2105/AJPH.2017.304044 29072941 PMC5922192

[37] Liu C, Yavar Z, Sun Q. Cardiovascular response to thermoregulatory challenges. Am J Physiol Heart Circ Physiol. 2015;309(11):H1793-H1812. doi:10.1152/ajpheart.00199.2015 26432837 PMC4698386

[38] Gostimirovic M, Novakovic R, Rajkovic J, . The influence of climate change on human cardiovascular function. Arch Environ Occup Health. 2020;75(7):406-414. doi:10.1080/19338244.2020.1742079 32200732

[39] Nawrot TS, Staessen JA, Fagard RH, Van Bortel LM, Struijker-Boudier HA. Endothelial function and outdoor temperature. Eur J Epidemiol. 2005;20(5):407-410. doi:10.1007/s10654-005-1068-x 16080588

[40] Guo Y, Barnett AG, Tong S. Spatiotemporal model or time series model for assessing city-wide temperature effects on mortality? Environ Res. 2013;120:55-62. doi:10.1016/j.envres.2012.09.001 23026801

