# Supplementary material for: Hourly Heat Exposure and Acute Ischemic Stroke
Source: JAMA Netw Open. 2024 Feb 28;7(2):e240627. doi: 10.1001/jamanetworkopen.2024.0627 (PMC10902723; doi:10.1001/jamanetworkopen.2024.0627)
Supplement: Supplement 1. — eMethods. Sensitivity Analysis eFigure 1. Flowchart for the Inclusion and Exclusion of AIS Patients eFigure 2. Geographic Distribution of the AIS Patients Included in This Study in China eFigure 3. National and Region-Specific Exposure-Response Curves for the Associations of AIS Onset With Extremely High Temperature From the Analysis With 3 Degrees of Freedom for the Natural Cubic Spline Applied in the Main Models eFigure 4. National and Region-Specific Lag Structures for the Associations of AIS Onset With Extremely High Temperature From the Analysis With the Maximum Lag of 36 Hours [file jamanetwopen-e240627-s001.pdf]

Supplemental Online Content

Zhu X, Chen R, Yuan J, et al. Hourly heat exposure and acute ischemic stroke. *JAMA Netw Open*. 2024;7(2):e240627. doi:10.1001/jamanetworkopen.2024.0627

eMethods. Sensitivity Analysis

eFigure 1. Flowchart for the Inclusion and Exclusion of AIS Patients

eFigure 2. Geographic Distribution of the AIS Patients Included in This Study in China

eFigure 3. National and Region-Specific Exposure-Response Curves for the Associations of AIS Onset With Extremely High Temperature From the Analysis With 3 Degrees of Freedom for the Natural Cubic Spline Applied in the Main Models

eFigure 4. National and Region-Specific Lag Structures for the Associations of AIS Onset With Extremely High Temperature From the Analysis With the Maximum Lag of 36 Hours

This supplemental material has been provided by the authors to give readers additional information about their work.

© 2024 Zhu X et al. *JAMA Network Open*

eMethods. Sensitivity Analysis

Methods

Multiple sensitivity analyses were conducted to evaluate the robustness of our findings by changing the degree of freedom (*df*) of the natural cubic spline in the cross-basis function (i.e., *df*=3), the maximum lag period (i.e., lag 36 h), and the radius of the weather station (i.e., within 50 km), as well as adjusting for each of five criteria air pollutants individually and all together over the same lag hours. Additionally, we calculated E-values of estimates of the national and region-specific associations to evaluate the potential influence due to the unobserved confounders [1]. Further, the subsequent temperature exposure after AIS onset was deemed as the negative control exposure due to the lack of causal links between future exposure and AIS onset [2,3], and its association with AIS onset was evaluated using the same model parameters in the main analysis. Finally, we derived the risk estimates of less extreme temperatures, including 95th percentile, 90th percentile, 80th percentile, 70th percentile, 60th percentile, and 50th percentile of temperature distribution.

Results

The results of sensitivity analyses (**Table 3 in the main text**) demonstrate that the main estimates remained stable after altering the *df* of cross-basis functions, restricting the distance between the home address and the nearest weather station within 50 km and adjusting for air pollutants. In **eFigure 3**, we found an absence of

the previously observed leveling-off in the exposure-response curve for the northern region after changing the *df* of the cross-basis function to three. Also, we observed a mild harvesting effect in the lag structures of 0 to 36 hours. The association was positive at lag 0 h, followed by a slightly negative association at lag 15 h—30 h, and returned to the null afterward (**eFigure 4**). The E-values for the estimates of the national and region-specific associations were 3.17 (Nationwide), 3.00 (North), and 2.52 (South), which indicates the observed associations were not likely to be influenced by unmeasured confounders [1]. There was a null association between future temperature exposure and AIS onset [OR: 1.23 (95%CI: 0.93, 1.95)] at the national level. The associations between high temperatures and AIS onset decreased by smaller quantiles of the temperature distribution. Notably, this association was consistently stronger in the northern region than in the southern region in all analyses using different definitions of high temperature. (**Table 3 in main text**).

References

[1] VanderWeele TJ, Ding P. Sensitivity Analysis in Observational Research: Introducing the E-Value. Ann Intern Med. 2017;167(4):268-74.

[2] Yu Y, Li H, Sun X, Liu X, Yang F, Hou L, et al. Identification and Estimation of Causal Effects Using a Negative-Control Exposure in Time-Series Studies With Applications to Environmental Epidemiology. Am J Epidemiol. 2021 Feb 1;190(3):468-476.

[3] Li Z, Lv S, Lu F, Guo M, Wu Z, Liu Y, et al. Causal Associations of Air Pollution With Cardiovascular

© 2024 Zhu X et al. *JAMA Network Open*

Disease and Respiratory Diseases Among Elder Diabetic Patients. Geohealth. 2023 Jun 20;7(6):e2022GH000730.

© 2024 Zhu X et al. *JAMA Network Open*

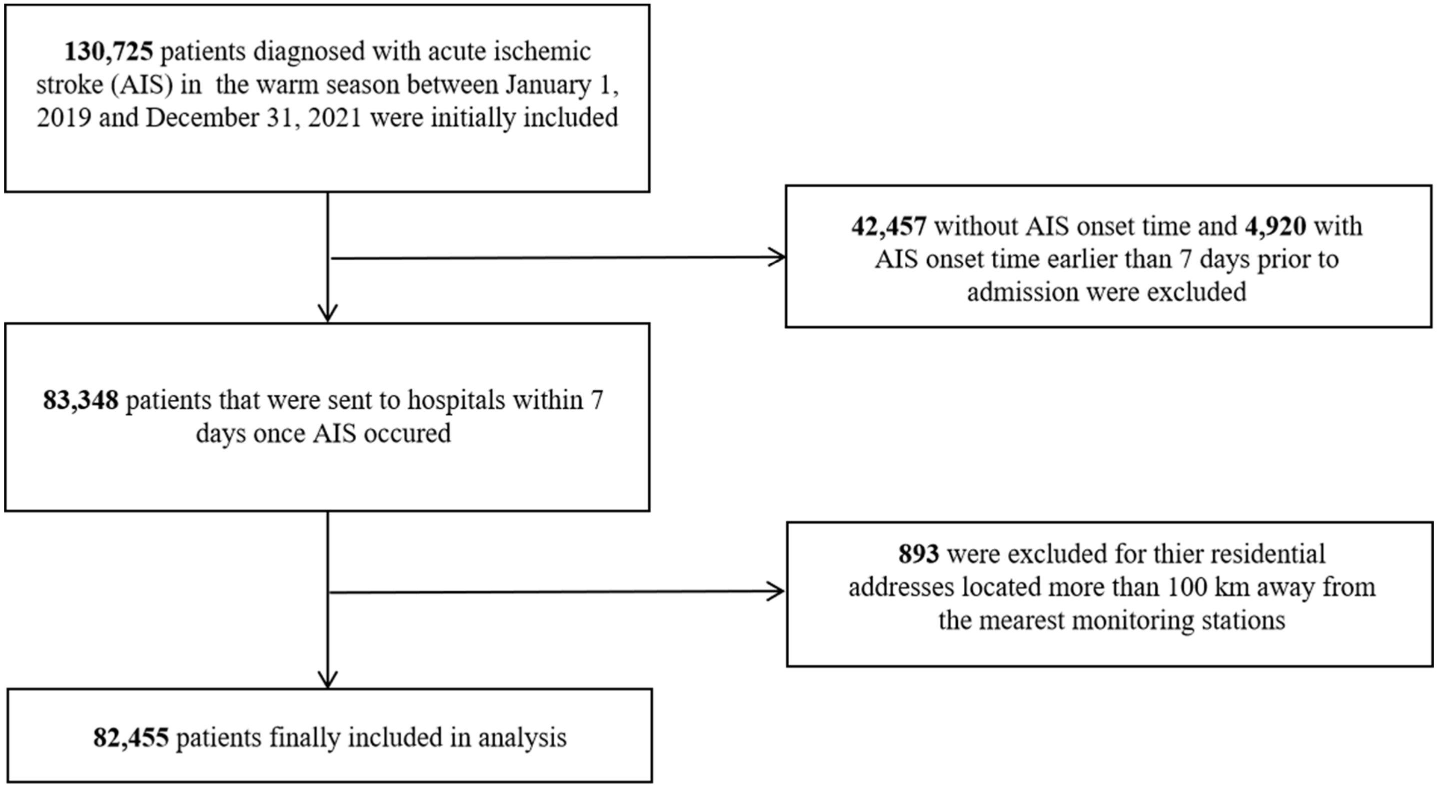

eFigure 1. Flowchart for the Inclusion and Exclusion of AIS Patients

© 2024 Zhu X et al. *JAMA Network Open*

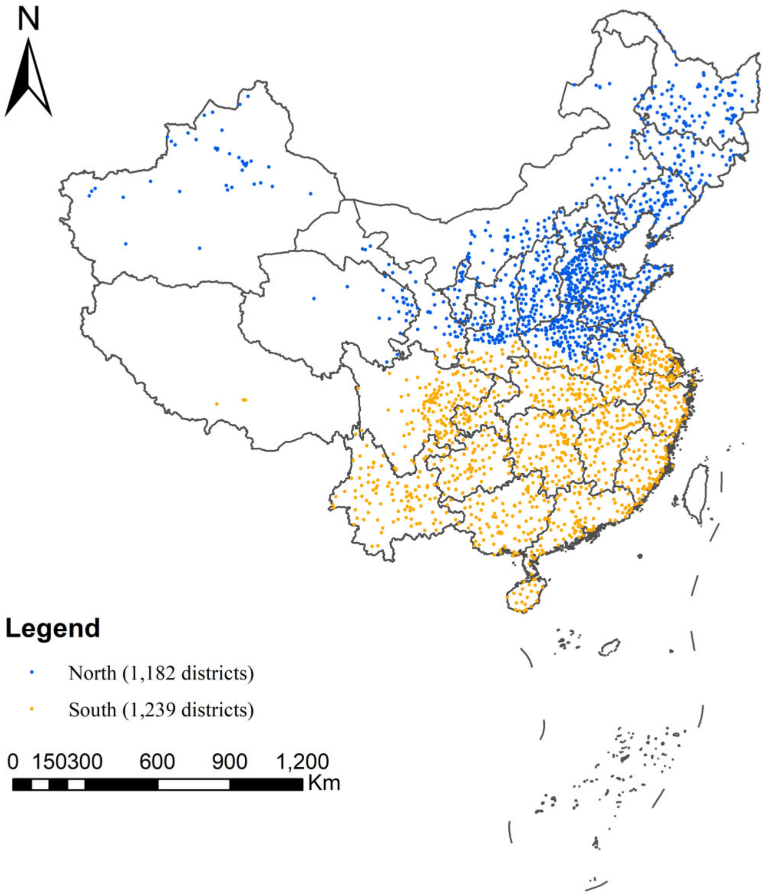

eFigure 2. Geographic Distribution of the AIS Patients Included in This Study in China

© 2024 Zhu X et al. *JAMA Network Open*

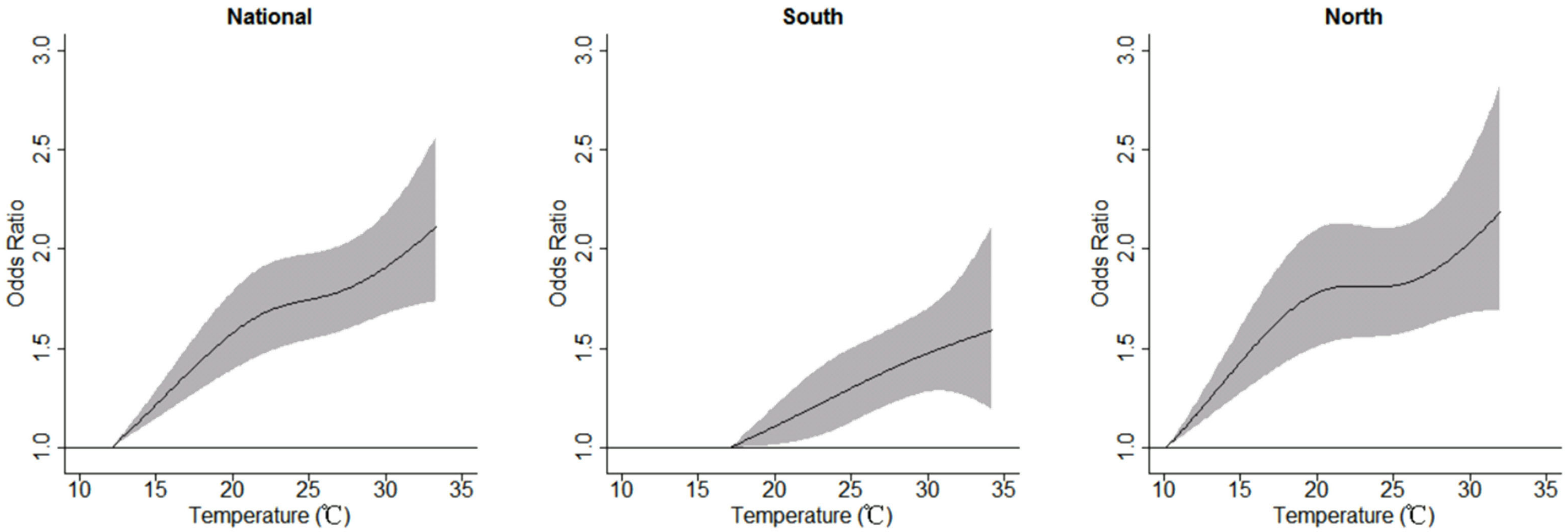

eFigure 3. National and Region-Specific Exposure-Response Curves for the Associations of AIS Onset With Extremely High Temperature From the Analysis

© 2024 Zhu X et al. *JAMA Network Open*

With 3 Degrees of Freedom for the Natural Cubic Spline Applied in the Main Models

© 2024 Zhu X et al. *JAMA Network Open*

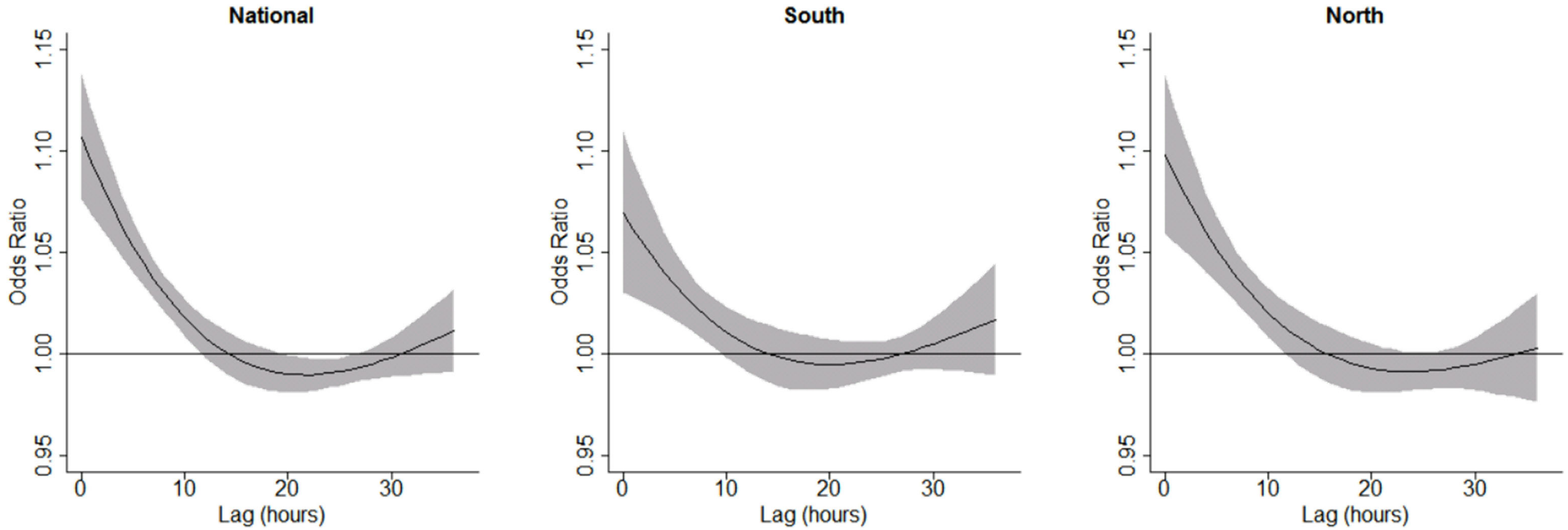

eFigure 4. National and Region-Specific Lag Structures for the Associations of AIS Onset With Extremely High Temperature From the Analysis With the Maximum Lag of 36 Hours

© 2024 Zhu X et al. *JAMA Network Open*
